# Comprehensive clinical benefit of CCM in HFrEF patients: a win-ratio analysis of FIX-HF-5C randomized trial

**DOI:** 10.1093/eschf/xvag179

**Published:** 2026-06-23

**Authors:** Rami Kahwash, Poying Lai, William T Abraham, Daniel Burkhoff, Rodrigo Chan, Andrew J Kaplan, Gery Tomassoni, Lee Ming Boo

**Affiliations:** Division of Cardiovascular Medicine, The Ohio State University Medical Center, 473W 12th Avenue, Suite 200, Columbus, OH 43210-1252, USA; Biostatistics, NAMSA (North American Science Associates, Inc.), St.Louis Park, MN 55426, USA; Division of Cardiovascular Medicine, The Ohio State University Wexner Medical Center and Davis Heart and Lung Research Institute, Columbus, OH 43210-1252, USA; Cardiovascular Research Foundation, NewYork, NY 10019, USA; Chan Heart Rhythm Institute, Mesa, AZ 85206, USA; Arizona Heart and Vascular, Glendale, AZ 85308, USA; Baptist Lexington, Lexington, KY 40503, USA; Clinical & Data Sciences, Impulse Dynamics, Marlton, NJ 08053, USA

**Keywords:** Cardiac Contractility Modulation (CCM), Composite endpoint, Heart Failure (HF), Left Ventricular Ejection Fraction (LVEF), NYHA

## Abstract

**Background:**

FIX-HF-5C multicentre, randomized study demonstrated cardiac contractility modulation (CCM^TM^) improved patient-centred outcomes, including functional capacity, symptom burden, and health-related quality of life. Reductions in cardiovascular death and heart failure hospitalization (HFH) were observed. These event-driven outcomes were not prespecified efficacy endpoints and were not incorporated into overall assessment of treatment benefit. Comprehensive clinical benefit of CCM therapy was re-analyzed by a win-ratio method that integrated event-driven clinical endpoints with patient-centred outcomes.

**Methods:**

The hierarchical win-ratio prioritized cardiovascular mortality, HFH, and patient-centred outcomes comprising equally weighted components of peak oxygen consumption, Minnesota Living with Heart Failure Questionnaire score, 6-minute walk distance, and NYHA functional class. Sub-group analyses were performed in patients with NYHA Class III versus IV.

**Results:**

In the primary analysis of 160 randomized patients (6364 patient pairs), the overall win ratio was 2.48 (95% CI 1.76 to 3.64; *P* < .001), corresponding to a 71% pairwise comparative probability of clinical benefit with CCM versus OMT. Event-driven clinical endpoints accounted for 22% of total wins while 56.2% came from patient-centric endpoints. CCM consistently outperformed OMT across all hierarchies. Sensitivity analyses yielded similar findings with win ratios favouring CCM (range: 1.52 to 2.42). Treatment effects were consistent across NYHA class III and IV subgroups.

**Conclusions:**

The win-ratio re-analysis supports and extends the original study results demonstrating a consistent clinical benefit of CCM therapy over OMT across a hierarchical framework integrating event-driven clinical endpoints with patient-centred functional outcomes. The findings, while clinically highly encouraging, are hypothesis-generating and warrant validation in future confirmatory studies.

## Graphical Abstract

**Graphical Abstract:**
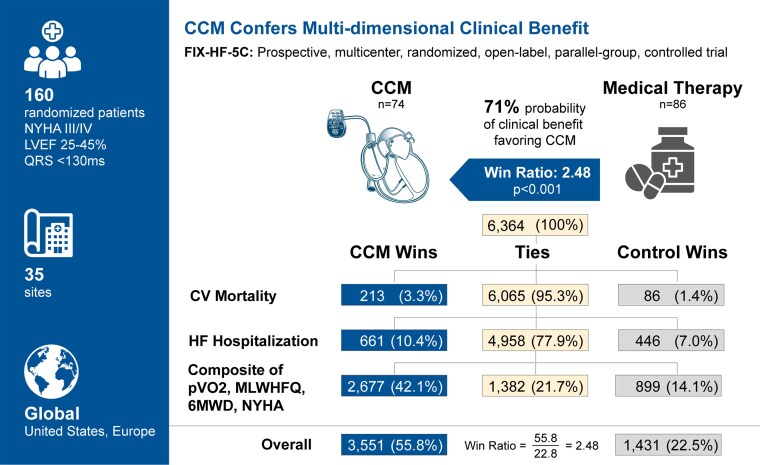

## Introduction

Cardiac contractility modulation (CCM™), delivered via the Optimizer™ Smart device, is an FDA-approved, device-based therapy that delivers high-voltage, non-excitatory electrical signals during the absolute refractory period to enhance myocardial contractility without increasing myocardial oxygen demand.^1,2^

Mechanistic and imaging studies have established a strong biological rationale for CCM, demonstrating favourable modulation of myocardial gene expression, improvements in global and regional left ventricular function, and promotion of reverse remodelling.^3–5^ Consistent with these mechanistic effects, early pilot and randomized clinical studies, supported by subsequent meta-analyses, demonstrated that CCM improves exercise capacity, functional status, and health-related quality of life in patients with symptomatic heart failure and reduced ejection fraction.^6–8^

FIX-HF-5C was a randomized confirmatory study that enrolled patients with NYHA class III—IVa symptoms, QRS duration <130 ms, and left ventricular ejection fraction (LVEF) 25% to 45%.^9^ The study demonstrated that CCM therapy significantly improved quality of life as measured by Minnesota Living With Heart Failure Questionnaire (MLWHFQ) score, NYHA functional class, and 6-minute walk distance (6MWD) while meeting prespecified safety criteria. These findings formed the basis for U.S. Food and Drug Administration approval of CCM therapy. Although exploratory analysis suggested a reduction in the composite of cardiovascular death and HF hospitalization, the trial was not powered to definitively assess event-based outcomes.

As heart failure therapeutics increasingly prioritize outcomes that matter most to patients, contemporary trials have adopted hierarchical composite endpoints and win-ratio methodologies to integrate mortality, heart failure hospitalizations, and functional measures within a clinically prioritized framework.^10^ This approach is particularly well suited for therapies such as CCM, whose principal benefits are expected to manifest through improvements in symptoms and functional capacity, with potential downstream effects on clinical events. Prior analyses have demonstrated that win-ratio–based methods yield conclusions concordant with traditional composite endpoints while providing greater granularity across multiple clinically meaningful domains.^11^

Accordingly, the objective of the present study was to reanalyze the FIX-HF-5C trial using a hierarchical win-ratio framework integrating mortality, heart failure hospitalizations, and validated patient-centred measures of symptoms and functional status to more comprehensively characterize the clinical benefit of CCM therapy.

## Method

The FIX-HF-5C study and its primary results have been published previously.^9^ Briefly, FIX-HF-5C was a prospective, unblinded, randomized, controlled trial comparing optimal medical therapy (OMT; control group) with OMT plus CCM (CCM; treatment group) in patients with NYHA class III or IVa HF, left ventricular ejection fraction (LVEF) 25% to 45%, and narrow QRS. All enrolled patients signed institutional review board approved informed consent forms. The study was conducted in compliance with the Declaration of Helsinki, national ethics and legal requirements. Effectiveness endpoints were evaluated at 24 weeks and included changes from baseline in pVO_2_, MLWHFQ score, NYHA functional class, and 6MWD. An independent clinical events committee of three cardiologists adjudicated all hospitalizations and deaths, including attribution to cardiovascular or HF-related causes.

### Win-ratio analysis

**Figure 1:**
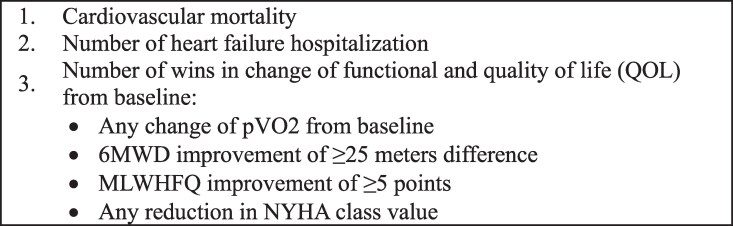
Hierarchical composite of clinical outcome components. Win-ratio analysis was conducted at three hierarchical levels as indicated. 6MWD, 6-minute walk distance; MLWHFQ, Minnesota Living With Heart Failure Questionnaire; NYHA, New York Heart Association; pVO_2_, peak oxygen consumption.

To evaluate the overall clinical benefit of CCM therapy, a win-ratio analysis was performed with three hierarchical levels, consisting of cardiovascular mortality, number of HF hospitalizations, and a combination of functional and QOL changes at 24 weeks from baseline (pVO_2_, 6MWD, MLWHFQ, NYHA) (*Figure 1*). At the third hierarchical level, all four components were assigned equal weight because there was no strong clinical or patient-preference evidence to support the superiority of any one outcome over the others. The ‘win’ was assigned to the patient showing more wins in the individual components. A meta-analysis performed recently indicated that a 25-meter increase in 6MWD was the minimum clinically important difference (MCID) to show improvements in pulmonary and cardiovascular conditions and was used in a similar win-ratio study.^12,13^ The patient with the greater quantitative change in 6MWD was assigned the win. If neither patient showed a minimum of 25-meter change, a tie was assigned. MLWHFQ measures the quality of life (QOL). The higher the MLWHFQ score, the poorer the QOL. A reduction of MLWHFQ indicates an improvement in QOL. A 5 or more points reduction (i.e. a change ≤−5 points) was considered clinically meaningful and has been used as the cut point in previous studies.^14,15^ The patient with a quantitatively greater score reduction was assigned the win. The patients were assigned a tie if the QOL score reduction was same or <5 points. No threshold was assigned to pVO2 in the primary analysis for 3 reasons: (a) this was the original study primary efficacy endpoint where no threshold was applied; (b) several measures were taken to minimize placebo effect including rigorous protocols with oversight of core laboratory (e.g. test results were read blinded, 2 tests on separate days were required); and (c) pVO2 is generally considered more objective than the other outcomes within the 3rd hierarchy (i.e. MLWHFQ, 6MWD, NYHA).

The primary analysis (Analysis 1) was based on intent-to-treat (ITT) population with 6364 possible patient pairs (74 CCM × 86 control). Six CCM-randomized patients were not implanted. A sensitivity analysis of the primary analysis was also performed with the per-protocol (PP, i.e. as-treated) population (68 CCM × 86 control = 5848 possible pairs).

### Sensitivity analysis

Three additional hierarchical win-ratio analyses (Analyses 2 to 4) were performed as sensitivity tests. Analysis 2 ranked individual functional and QOL parameters hierarchically after cardiovascular death and HF hospitalization (Supplementary Table S1). The pVO_2_ was ranked third because it was the original trial’s primary efficacy endpoint. A ≥6% increase in pVO_2_ was implemented for this analysis as this value was considered clinically meaningful in reduction of cardiovascular mortality or heart failure hospitalization^16^ and that a threshold for pVO_2_ is needed to allow for incorporation of other efficacy endpoints of lower hierarchy. The patient with higher percentage change wins. If neither of a patient-pair had a 6% increase, a tie was assigned. MLWHFQ was ranked fourth given its superior sensitivity to clinical change compared with NYHA and 6MWD.^17,18^ The 6MWD was ranked higher than NYHA due to its greater objectivity in measurements.

Analysis 3 repeated the primary win-ratio analysis but did not require minimum thresholds for 6MWD or MLWHFQ changes (Supplementary Table S2). Wins were assigned solely based on the relative magnitude of change from baseline.

Analysis 4 ranked cardiovascular mortality, HF hospitalization, and pVO_2_ as the first three hierarchical levels, due to pVO_2_ being the primary efficacy endpoint of the study (Supplementary Table S3). The remaining three functional/QOL parameters (6MWD, MLWHFQ, NYHA class) were grouped at the final level, with the win assigned to the patient achieving more wins of these components.

Analysis plan for the primary analysis and all sensitivity analyses, including the hierarchies and thresholds, were pre-specified before the win-ratio analyses were executed.

### Statistical methods

Continuous variables were summarized as mean ± SD, and categorical variables as counts and percentages. Between groups comparisons of *Table 1* were performed using independent samples t-tests, Fisher’s exact test or Chi-square test, as appropriate. The primary analysis used the *win-ratio* unmatched pair approach to compare clinical outcomes between the CCM and control groups. Each CCM-treated patient was paired with each control patient, and pairs were evaluated hierarchically according to: (1) cardiovascular mortality, (2) number of heart-failure hospitalizations, and (3) changes from baseline to 24 weeks in functional and quality-of-life measures (pVO_2_, 6MWD, MLWHFQ score, and NYHA class). Wins and ties were determined at each hierarchical level using prespecified clinically meaningful thresholds. The win ratio was calculated as the total number of wins in CCM group divided by wins in control group. Missing data was not imputed. Any missing data within a comparison pair was treated as a tie. Within the primary analysis level 3 composite, comparisons were performed only when both patients within a comparison pair had data for all the 4 components. Statistical significance was assessed using the Finkelstein & Schoenfeld method. Three sensitivity analyses were performed that varied the hierarchical ordering of components or removed minimal clinically important thresholds. Additionally, a sensitivity analysis was performed using the PP population, i.e. as-treated, for the primary Win Ratio analysis. Subgroup analyses were conducted stratified by baseline NYHA class (III vs IV). Estimation of 95% confidence intervals (CIs) were made via bootstrap resampling (500 iterations) for each subgroup. Interaction tests were performed by comparing the log (win ratios) and their standard errors across subgroups. Two-sided *P*-values <.05 were considered statistically significant. Analyses were conducted using SAS Life Sciences Analytics Framework (LSAF). All statistical code and results were independently validated.

**Table 1:** Proportion of patients or rate of events between two study groups (ITT population)

Endpoint	CCM	Control	*P*-value
Total	74	86	
CV Mortality or Any HF Hospitalization, n/N (%)	7/74 (9.5%)	13/86 (15.1%)	.2807**
CV Mortality, n/N (%)	1/74 (1.4%)	3/86 (3.5%)	.6246*
Any HF hospitalization, n/N (%)	6/74 (8.1%)	11/86 (12.8%)	.3379**
Number of HF hospitalization, mean ± SD (N)	0.1 ± 0.39 (74)	0.2 ± 0.46 (86)	.4209***
Rate of HF hospitalization, n/10 years, mean ± SD (N)^a^	0.6 ± 2.46 (71)	3.1 ± 21.91 (86)	.3537***
pVO_2_, n/N (%)			
1| Improved (≥+6% from baseline)	25/68 (36.8%)	20/71 (28.2%)	.5549**
2| Unchanged (0 to <+6% from baseline)	9/68 (13.2%)	11/71 (15.5%)	
3| Worsened (<0 from baseline)	34/68 (50%)	40/71 (56.3%)	
MLWHFQ, n/N (%)			
1| Improved (≤−5 from baseline)	54/70 (77.1%)	45/76 (59.2%)	.0501**
2| Unchanged (0 to >−5 from baseline)	6/70 (8.6%)	8/76 (10.5%)	
3| Worsened (>0 from baseline)	10/70 (14.3%)	23/76 (30.3%)	
6MWD, n/N (%)			
1| Improved (≥+25 m from baseline)	45/69 (65.2%)	31/72 (43.1%)	.0151**
2| Unchanged (0 to <+25 m from baseline)	10/69 (14.5%)	11/72 (15.3%)	
3| Worsened (<0 from baseline)	14/69 (20.3%)	30/72 (41.7%)	
NYHA, n/N (%)			
1| Improved (lower class)	57/70 (81.4%)	32/75 (42.7%)	<.0001**
2| Unchanged (same class)	13/70 (18.6%)	42/75 (56%)	
3| Worsened (higher class)	0/70 (0%)	1/75 (1.3%)	

Note: n, number of patients with event; N, number of patients participated in the observation.

^a^Rate of HF hospitalization is calculated as the number of hospitalizations divided by days to censoring, scaled to 10 years.

^*^ *P*-value from Fisher’s exact test.

^**^ *P*-value from Chi-square test.

^***^ *P*-value from independent samples *t*-test.

## Results

A total of 160 patients were randomized, 86 to the control group and 74 to the CCM group. Of the 74 patients assigned to CCM, 68 were successfully implanted and received CCM therapy. At 24 weeks, 146 patients completed the study (79 control, 67 CCM). Baseline demographic and clinical characteristics, including age, sex, comorbidities, LVEF, 6MWD, MLWHFQ score, and NYHA class, were comparable between groups and have been reported previously.^9^ Unlike the original FIX-HF-5C trial report, subjects with EF 25% to 45% from the FIX-HF-5 trial was not included.

*Table 1* summarizes the proportions of patients achieving meaningful clinical changes or experiencing clinical events. Functional and quality-of-life outcomes strongly favoured CCM therapy: a significantly greater proportion of CCM-treated patients achieved clinically meaningful improvements in MLWHFQ score (≥5-point reduction) and 6MWD (≥25-m increase). Similarly, a higher proportion of CCM-treated patients improved in NYHA class. Although cardiovascular mortality, HF hospitalization, and their composite occurred less frequently in the CCM group, these differences were not statistically significant using traditional analyses due to low events rate and that the study was not originally powered for these endpoints.

### Primary analysis

**Figure 2:**
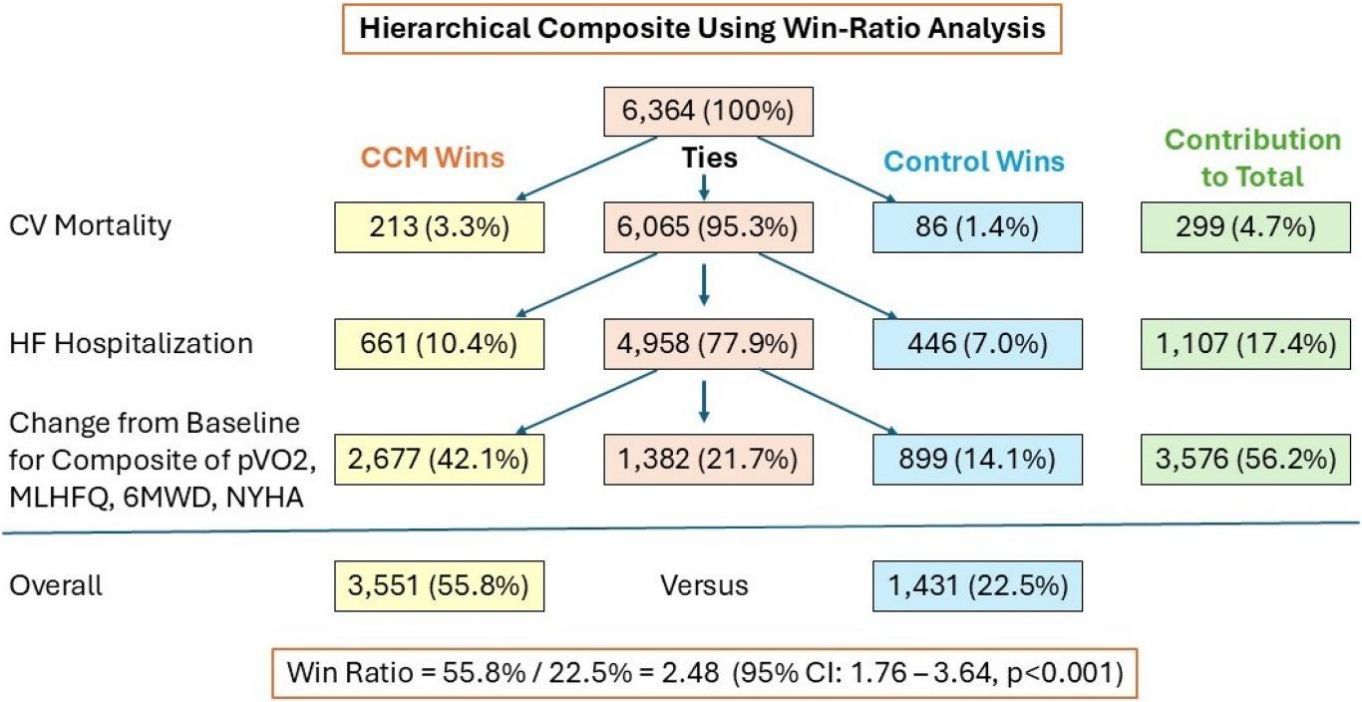
Hierarchical composite using win-ratio analysis. A total of 6364 patient-pairs from FIX-HF-5C study were examined for CCM Wins, Ties and Control Wins at each hierarchical levels. The win-ratio was calculated based on total number of CCM wins and control wins. CCM, cardiac contractility modulation.

To comprehensively reassess overall treatment effects, cardiovascular mortality and HF hospitalization were prioritized as the top two levels of the hierarchical win-ratio analysis (*Figure 1*). *Figure 2* displays win ratios for individual endpoints and the overall win ratio by following analysis procedures described previously.^19^ The primary analysis yielded an overall win ratio of 2.48 (95% CI 1.76 to 3.64; *P* < .001), significantly favouring CCM therapy. This corresponds to an estimated 71% (2.48/(1 + 2.48)) pairwise comparative probability that a randomly selected CCM-treated patient would have a more favourable clinical outcome than a control patient.

In this primary win-ratio analysis, CV mortality and HF hospitalization contributed 22% (4.7% + 17.4%) to the total wins, with CCM contributed 13.7% and control 8.4%. The composite of pVO_2_, 6MWD, MLWHFQ and NYHA accounted for the remaining 56% of wins. Parameter-specific win percentages are provided in *Table 2*. Across all domains, CCM therapy generated a higher proportion of wins, with the largest contribution coming from quality-of-life (MLWHFQ) improvement. Ties for CV mortality and HF hospitalization were primarily due to ties in event counts; there were no ties due to missing data. Ties at the composite level 3 were due to missing data in any components (55.9%, 772/1382) or same wins and losses counts (44.1%, 610/1382).

**Table 2:** Primary win-ratio analysis for individual parameters

Endpoint component	CCM win	Control win	Win ratio (CCM/Control wins)	Contribution to total	Tie
CV Mortality	213 (3.3%)	86 (1.4%)	2.48	299 (4.7%)	6065 (95.3%)
HF hospitalization	661 (10.4%)	446 (7.0%)	1.48	1107 (17.4%)	4958 (77.9%)
Number of Win Based on composite of four parameters	2677 (42.1%)	899 (14.1%)	2.98	3576 (56.2%)	1382 (21.7%)
Any pVO_2_ change from baseline	1859 (52.0%)	1400 (39.1%)			
6MWD Improvement by ≥25 metres	1914 (53.5%)	876 (24.5%)			
MLWHFQ improvement by ≥5 pts	2301 (64.3%)	887 (24.8%)			
Any reduction NYHA class value	1859 (52.0%)	1400 (39.1%)			
Total	3551 (55.8%)	1431 (22.5%)	2.48 (95% CI: 1.76 to 3.64, *P* < .001)		1382 (21.7%)

### Sensitivity analyses

Three sensitivity analyses were performed using variations of the hierarchical endpoints (*Table 3*), which yielded similar results, confirming the robustness of the clinical effects of CCM (Supplementary Tables S1—S3). Additional sensitivity analyses were performed with the PP population which yielded similar results but slightly higher win-ratio of 2.66 (*P* < .001) (Supplementary Table S4).

**Table 3:** Sensitivity analyses using variations of the hierarchical endpoints

Hierarchy	Analysis 2	Analysis 3	Analysis 4
1	CV Mortality	CV Mortality	CV Mortality
2	HF hospitalization	HF hospitalization	HF hospitalization
3	pVO2 (≥6%)	Composite of four parameters: pVO2, MLWHFQ, 6MWD, NYHA (any improvement wins)	pVO2 (≥6%)
4	MLWHFQ (≥5pt improvement)		Composite of three parameters: MLWHFQ (≥5pt improvement), 6MWD (≥25 m), NYHA:
5	6MWD (≥25 m)		
6	NYHA		
Win Ratio	1.52 (95% CI: 1.07 to 2.15, *P* = .01)	2.42 (95% CI: 1.72 to 3.61, *P* < .001)	1.74 (95% CI: 1.21 to 2.65, *P* = .003)

### Subgroup analysis by NYHA class

**Figure 3:**
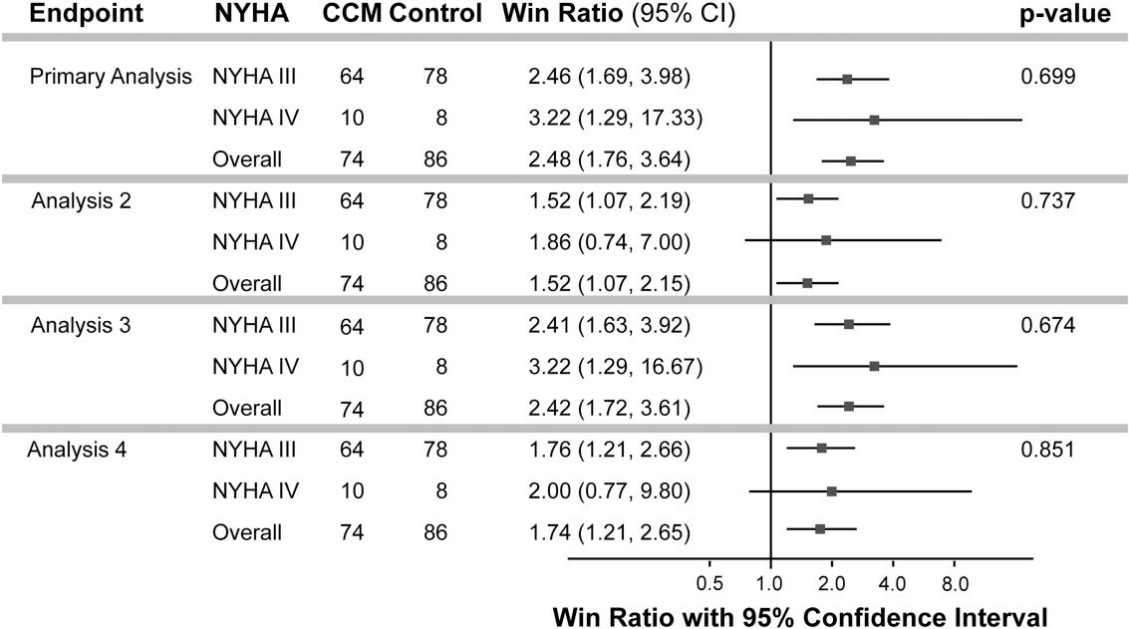
Stratified Win-Ratio Analyses in NYHA Class III and IV Patients. Left: win-ratio analyses were performed by following the procedures described in method sections for Primary Analysis and Analysis 2 to 4 based on NYHA class III and IV strata and overall population. Numbers under CCM and Control are total wins for individual strata and analyses. Right: graphical presentation of win-ratios and 95% confidence intervals (95% CI). *P*-value represents comparison between NYHA III and IV cohorts. CCM, cardiac contractility modulation; NYHA, New York Heart Association.

Subgroup analysis of the earlier CCM trial (Fix-HF-5) suggested that treatment benefit with CCM was greater in subjects with NYHA class III than IV.^8^ Patients in Fix-HF-5C study were therefore stratified into NYHA class III and IV and the four win-ratio analyses were conducted in each stratum (*Figure 3*). In the primary analysis, overall win-ratios were 2.46 for NYHA class III and 3.22 for NYHA class IV, both significantly favouring CCM. As expected, the 95% CI was wide in NYHA class IV due to small sample size, but no statistically significant difference was observed between NYHA III and IV (*P* = .699).

Results for NYHA III patients closely mirrored those of the overall population across all analyses. In NYHA IV patients, although primary analysis win ratio favoured CCM, confidence interval was wide and some sensitivity analyses (i.e. analyses 2 and 4) did not reach statistical significance due to small sample size.

## Discussion

In this re-analysis of FIX-HF-5C using a hierarchical win-ratio framework, we performed a more comprehensive assessment of clinical efficacy integrating mortality, HF hospitalization, functional capacity, and QOL outcomes. Across all analytic approaches, including primary and sensitivity analyses, CCM therapy consistently demonstrated favourable clinical effects in patients with NYHA class III and IV HF. Specifically in the primary analysis, CCM therapy was associated with a 71% estimated pairwise comparative probability of clinical benefit compared with control (OMT), reflecting a robust and directionally consistent pattern of wins across the entire clinical hierarchy. These observations strengthened the conclusion of the overall positive clinical effectiveness of CCM in not only symptomatic and functional improvements but also points to the potential benefit in event outcomes such as mortality and HF hospitalization.

Prior studies have shown that CCM therapy improves QOL, functional capacity, and cardiac remodelling,^5^ consistent with current findings that patient-centric endpoints contributed to 56.2% of wins with greater favourability towards CCM (CCM 42.1% vs control 14.1%). Although FIX-HF-5C was not powered for detection of improvements in cardiovascular mortality or HF hospitalization, these event outcomes contributed meaningfully to the overall win ratio. Event components accounted for 22% of the total wins, with results numerically favouring CCM (CCM 13.4% vs control 8.4%; win ratio 1.59). These findings are directionally concordant with the original FIX-HF-5C report, in which CCM improved survival free of cardiac death and HF hospitalization (97.1% vs 89.2%; *P* = .048). Few other contemporary HF trials had used win-ratio methods. In the EMPULSE trial, empagliflozin demonstrated mortality/HF event wins of 18.3% vs 12.2% (win ratio 1.5).^19^ In contrast, the BeAT-HF study showed a neutral effect on CV death/HF events (30% vs 29%; win ratio 1.03).^20^ Larger randomized trials will be needed to definitively determine whether CCM therapy favourably affects these hard clinical outcomes.

Stratified analyses demonstrated that the win-ratio estimates for NYHA class III and IV patients were similar in the primary analysis, definitive clinical benefit in NYHA IV population is not possible due to small sample size. These findings should be interpreted as hypothesis-generating but support the rationale for future confirmatory studies of CCM therapy in NYHA class IV patients.

Traditional HF trial designs require selecting either symptoms or time-to-event outcomes as the primary endpoint, each with inherent limitations. As guideline-directed medical therapy continues to improve event-free survival, detecting incremental benefits using traditional time-to-first-event analyses requires prohibitively large sample sizes and may overlook patient-centred benefits such as symptoms and functional status. Conversely, trials focused solely on symptomatic endpoints may not fully capture the therapy’s impact on clinical events. The win-ratio methodology provides an integrated, hierarchy-based approach that accounts for the relative clinical importance of disparate outcomes and allows a more holistic assessment of therapeutic benefit. When interpreted appropriately, win-ratio analyses can offer a comprehensive evaluation of therapies whose benefits span symptoms, function, and clinical events. Our findings suggest that this framework is particularly well suited for therapies such as CCM, where multidimensional benefits may not be fully captured by conventional analytic methods.

### Limitations

Although the win-ratio framework enables assessment of clinically meaningful benefit across a hierarchy of event-based, symptomatic, and functional outcomes, several limitations should be considered when interpreting these findings:

- **Post-hoc nature of the analysis:**
  This was a post-hoc evaluation and should therefore be regarded as hypothesis generating. The findings require confirmation in prospectively designed studies using hierarchical methodologies.
- **Residual ties inherent to the win-ratio method:**
  The win-ratio approach can leave a proportion of patient pairs unresolved when both subjects tie across the full hierarchy of outcomes. In our primary analysis, approximately 21% of pairwise comparisons remained tied. Because all major effectiveness endpoints available in the FIX-HF-5C dataset were incorporated, additional minor outcomes were unlikely to meaningfully reduce these ties. Importantly, the consistent directionality observed across all secondary and sensitivity analyses supports the robustness of the favourable win ratio for CCM.
- **Lack of temporal information:**
  Unlike traditional time-to-event analyses, the win ratio does not convey when clinical events occurred. However, previously published Kaplan–Meier analyses from FIX-HF-5C demonstrated that results consistent with the current findings, including lower rates of cardiovascular mortality or HF hospitalization with CCM (CCM 2.9% vs control 10.8%; *P* = .042).^9^
- **Lack of sham-control arm**
  FIX-HF-5C was not sham-controlled or blinded. Potential placebo-effect or variability in responses/measurements existed for endpoints such as MLWHFQ, 6MWD and NYHA, which contributed to majority of the wins in this analysis.

### Conclusion

The win-ratio re-analysis from FIX-HF-5C supports and extends the original study results demonstrating the clinical benefits of CCM therapy when outcomes are evaluated across a hierarchical framework integrating mortality, HF events, symptoms, functional capacity, and QOL. The overall win ratio of 2.48, corresponding to a 71% estimated pairwise comparative probability of a win for patients receiving CCM over OMT alone. Cardiovascular mortality and HF hospitalization contributed 22% of total wins, underscoring the clinical relevance of the observed treatment effect. Favourable and directionally consistent effects at every level of the hierarchical analysis—as well as across all secondary analyses—reinforce the robustness of these findings. The findings, while clinically highly encouraging, are hypothesis-generating and warrant validation in future confirmatory studies.

## Supplementary Material

xvag179_Supplementary_Data

## Supplementary data

### Data Availability

The data underlying this article cannot be shared publicly due to privacy of individuals that participated in the study. The derived data generated in this research are available in the article and in its online Supplementary material.

## Declarations

### Pre-registered Clinical Trial Number

None supplied.
