# Supplementary material for: Comprehensive clinical benefit of CCM in HFrEF patients: a win-ratio analysis of FIX-HF-5C randomized trial
Source: ESC Heart Fail. 2026 Jun 23;13(4):xvag179. doi: 10.1093/eschf/xvag179 (PMC13367581; doi:10.1093/eschf/xvag179)
Supplement: xvag179_Supplementary_Data [file xvag179_supplementary_data.docx]

**Online-Only Supplementary Materials: Supplemental Tables S1-S4**

**Supplemental Table S 1: Analysis 2 - Win-Ratio Analysis Based on Six Hierarchical Levels**

| **Hierarchical Parameter** | **CCM Win** | **Control Win** | **Win Ratio (CCM / Control Wins)** | **Contribution to Total** | **Tie** |
| --- | --- | --- | --- | --- | --- |
| Cardiovascular Mortality | 213  (3.3%) | 86  (1.4%) | 2.48 | 299  (4.7%) | 6065  (95.3%) |
| Number of HF Hospitalization | 661  (10.4%) | 446  (7.0%) | 1.48 | 1107  (17.4%) | 4958  (77.9%) |
| pVO_2_ Change from Baseline by ≥6% | 1256  (19.7%) | 1034  (16.2%) | 1.21 | 2290  (36.0%) | 2668  (41.9%) |
| MLWHFQ Improvement by ≥ 5 points | 1132  (17.8%) | 594  (9.3%) | 1.91 | 1726  (27.1%) | 942  (14.8%) |
| 6MWD Improvement by ≥25 Meters | 109  (1.7%) | 61  (1.0%) | 1.79 | 170  (2.7%) | 772  (12.1%) |
| Any Reduction in NYHA Class Value | 34  (0.5%) | 13  (0.2%) | 2.62 | 47  (0.7%) | 725  (11.4%) |
| Total | 3405  (53.5%) | 2234  (35.1%) | 1.52  (95% CI: 1.07-2.15, p = 0.01) |  | 725  (11.4%) |

**Supplemental Table S 2: Analysis 3 - Win-Ratio Analysis with Clinical and QOL Parameter Changes without A Threshold Value**

| **Endpoint Component** | **CCM Win** | **Control Win** | **Win Ratio (CCM / Control Wins)** | **Contribution to Total** | **Tie** |
| --- | --- | --- | --- | --- | --- |
| Cardiovascular Mortality | 213  (3.3%) | 86  (1.4%) | 2.48 | 299  (4.7%) | 6065  (95.3%) |
| Number of HF Hospitalization | 661  (10.4%) | 446  (7.0%) | 1.48 | 1107  (17.4%) | 4958  (77.9%) |
| Number of Wins Based on Four Parameters | 2603  (40.9%) | 902  (14.2%) | 2.89 | 3505  (55.1%) | 1453  (22.8%) |
| Any pVO_2_ change from baseline | 1838  (52.4%) | 1346  (38.4%) |  |  |  |
| Any 6MWD improvement | 2187  (62.4%) | 1078  (30.8%) |  |  |  |
| Any MLWHFQ improvement | 2459  (70.2%) | 991  (28.3%) |  |  |  |
| Any reduction in NYHA class value | 2026  (57.8%) | 368  (10.5%) |  |  |  |
| Total | 3477  (54.6%) | 1434  (22.5%) | 2.42 (95% CI: 1.72 – 3.61, p <0.001) |  | 1453  (22.8%) |

**Supplemental Table S 3: Analysis 4 – Win-Ratio Analysis with pVO2 at Third Hierarchical Level**

| **Endpoint Component** | **CCM Win** | **Control Win** | **Win Ratio (CCM / Control Wins)** | **Contribution to Total** | **Tie** |
| --- | --- | --- | --- | --- | --- |
| Cardiovascular Mortality | 213  (3.3%) | 86  (1.4%) | 2.48 | 299  (4.7%) | 6065  (95.3%) |
| Number of HF hospitalization | 661  (10.4%) | 446  (7.0%) | 1.48 | 1107  (17.4%) | 4958  (77.9%) |
| pVO_2_ change from baseline by ≥6% | 1256  (19.7%) | 1034  (16.2%) | 1.21 | 2290  (36.0%) | 2668  (41.9%) |
| Number of Wins Based on Four Parameters | 1292  (20.3%) | 399  (6.3%) | 3.24 | 1691  (26.6%) | 977  (15.4%) |
| 6MWD improvement by ≥25 meters | 910  (53.8%) | 417  (24.7%) |  |  |  |
| MLWHFQ improvement by ≥5pts | 1063  (62.9%) | 445  (26.3%) |  |  |  |
| Any reduction in NYHA class value | 1124  (66.5%) | 188  (11.1%) |  |  |  |
| Total | 3422 (53.8%) | 1965  (30.9%) | 1.74 (95% CI: 1.21 – 2.65, p = 0.003) |  | 977  (15.4%) |

**Supplemental Table S 4: Primary Win-Ratio Analysis for Per Protocol Population**

| **Endpoint Component** | **CCM Win** | **CONTROL Win** | **Win Ratio (CCM / CONTROL Wins)** | **Contribution to Total** | **Tie** |
| --- | --- | --- | --- | --- | --- |
| CV Mortality | 204 (3.5%) | 0 (0.0%) | . | 204 (3.5%) | 5644 (96.5%) |
| HF hospitalization | 627 (10.7%) | 446 (7.6%) | 1.41 | 1073 (18.3%) | 4571 (78.2%) |
| Change from baseline comparison (4 variables) | 2576 (44.0%) | 835 (14.3%) | 3.09 | 3411 (58.3%) | 1160 (19.8%) |
| pVO_2_ (any difference) | 1795 (52.6%) | 1357 (39.8%) |  |  |  |
| 6MWD (+≥25m) | 1861 (54.6%) | 836 (24.5%) |  |  |  |
| MLWHFQ (- ≥5pts) | 2219 (65.1%) | 828(24.3%) |  |  |  |
| NYHA (any difference) | 2089 (61.2%) | 369 (10.8%) |  |  |  |
| Total | 3407 (58.3%) | 1281 (21.9%) | 2.66 p = <0.001 |  | 1160 (19.8%) |
